# Metastatic Adenocarcinoma of Temporal Bone with Collet-Sicard Syndrome

**Published:** 2018-11

**Authors:** Sethu T Subha, Abdul-Jalil Nordin

**Affiliations:** 1 *Department of Otorhinolaryngology, University Putra Malaysia (UPM), Selangor, Malaysia.*

**Keywords:** Adenocarcinoma, Collet-Sicard syndrome, Cranial nerve palsies, Computed tomography, 18F-FDG PET-CT (18 Fluorodeoxyglucose Positron Emission Tomography Computerised Tomography), metastasis, Temporal bone

## Abstract

**Introduction:**

Metastatic tumors of the temporal bone are extremely rare. Collet-Sicard syndrome is an uncommon condition characterized by unilateral palsy of the lower four cranial nerves. The clinical features of temporal bone metastasis are nonspecific and mimic infections such as chronic otitis media and mastoiditis.

**Case Report:**

This report describes a rare case of metastatic adenocarcinoma of the temporal bone causing Collet-Sicard syndrome, presenting with hearing loss, headache and ipsilateral cranial nerve palsies. The patient was a 68-year old woman initially diagnosed with extensive mastoiditis and later confirmed as having metastatic adenocarcinoma of the temporal bone, based on histopathologic findings.

**Conclusion:**

Clinical presentation of metastatic carcinoma of the temporal bone can be overshadowed by infective or inflammatory conditions. This case report is to emphasize the point that a high index of clinical suspicion is necessary for the early diagnosis of this aggressive disease which carries relatively poor prognosis. This report highlights that it is crucial to suspect malignant neoplasm in patients with hearing loss, headache and cranial nerve palsies.

## Introduction

Metastatic tumors of the temporal bone are relatively uncommon and usually originate from the sites with a tendency to metastasize to bone (1). The most common areas of temporal bone metastasis are the petrous apex internal auditory canal and mastoid (1,2). The clinical features of temporal bone metastasis are nonspecific and can mimic infections such as chronic suppurative otitis media and mastoiditis (2,3). The rare entity Collet-Siccard syndrome involves palsy of the glossopharyngeal, vagus, accessory and hypoglossal nerves (4). The common causes of this syndrome are trauma, vascular diseases and tumors in the skull base and nasopharynx (4,5). 

## Case Report

A 68-year-old woman presented with headache, hearing loss, facial asymmetry and dysphagia for few months. On examination, the patient had right facial palsy (grade IV based on the House-Brackmann scale). Otoscopic and nasal endoscopic examinations were found to be normal. Examination of the oropharynx and larynx revealed right-sided 9th, 10th and 12th cranial nerve palsies with loss of the gag reflex, paralysis of the soft palate, paralysis of the right hypoglossal palsy with deviation of tongue to the right side and right-sided vocal cord palsy. Neck examination showed drooping of the right shoulder and there were no palpable neck nodes. Pure-tone audiogram demonstrated right-sided mixed hearing loss. 

**Fig 1:**
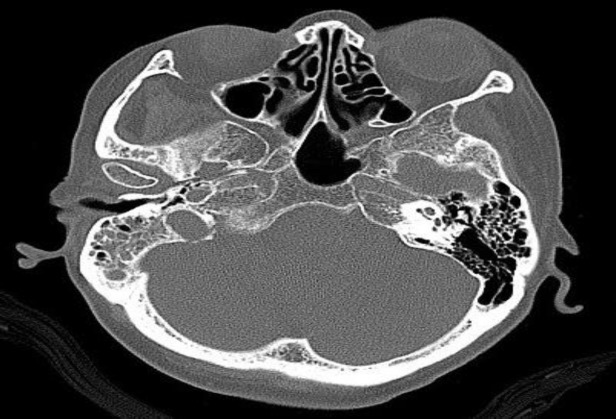
CT temporal bone showing lesions in the mastoid with sclerosis in the basiocciput

**Fig 2:**
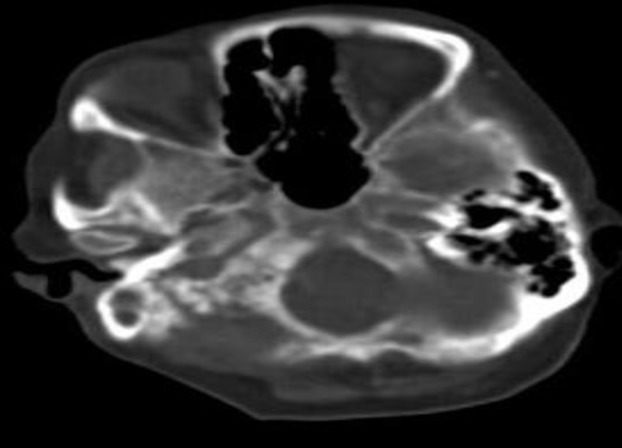
CT temporal bone showing a soft tissue density lesion in the right mastoid air cells with sclerosis. The sclerosis extends into the basiocciput and lateral part of the clivus

**Fig 3:**
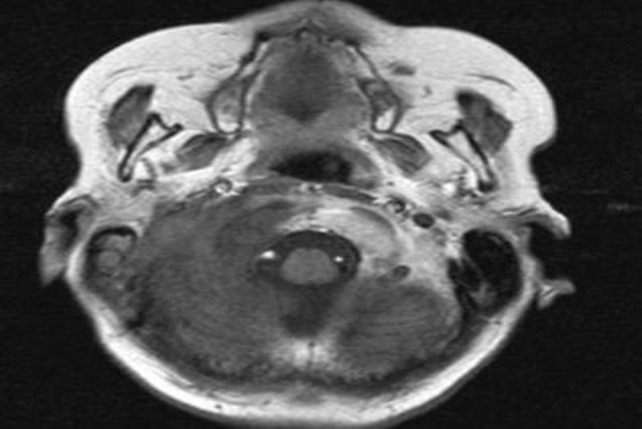
MRI of the brain. Axial T1 weighted sequence demonstrates loss of normal signal intensity over the right mastoid, and the heterogenous lesion in the medial part of the mastoid extends to the petrous apex with involvement of the clivus

 A computed tomography (CT) scan and contrast enhanced magnetic resonance imaging (MRI) showed soft tissue lesion in the mastoid with sclerosis in the basiocciput extending to the clivus and sphenoid; features suggestive of extensive mastoiditis with cholesteatoma (Fig.1–3). The bony changes in the occipital bone could possibly be due to fibrous dysplasia or metastases. The patient underwent biopsy and debulking of the tumor, and histopathological examination confirmed it to be a metastatic adenocarcinoma.

**Fig 4:**
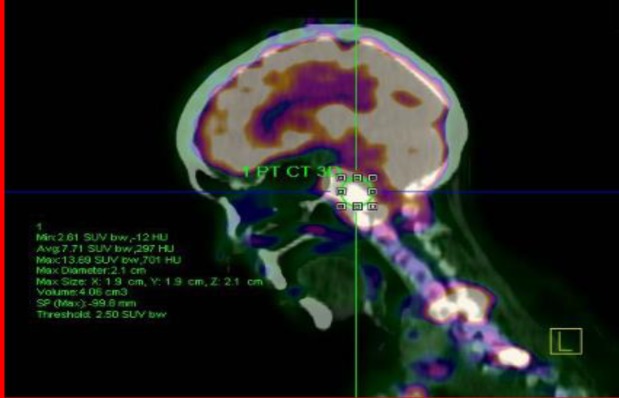
Sagittal reconstructed 18F-FDG PET-CT showing multiple bony involvement ,with the skull base including the right petrous bonebone with intracranial extension and multilevel axial skeleton involvement including the upper cervical spine (C1 to C4), dorsal vertebral (D11)

**Fig 5:**
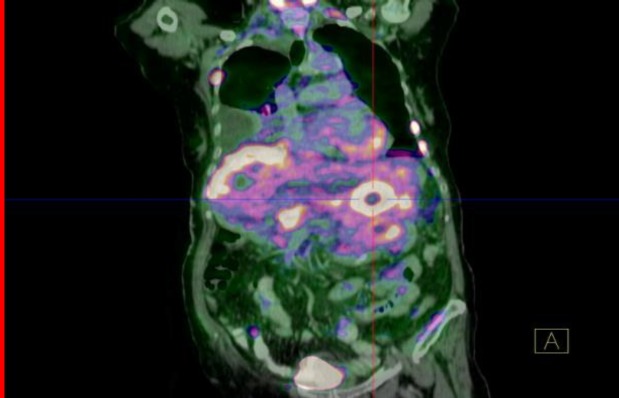
Coronal reconstructed 18F-FDG PET-CT image of the abdomen showing multifocal areas with intense FDG uptake indicating extensive liver and subdiaphragmatic involvement.The lesion in the liver demonstrate peculiar ‘doughnut ‘ appearance indicating centrally located necrotised parenchyma

Positron Emission Tomography CT using 18-fluorodeoxyglucose (^18^F-FDG PET-CT) tracer demonstrated multiple bony involvement, with the skull base including the right petrous bone with intracranial extension and multilevel axial skeleton involvement including the upper cervical spine (C1 to C4), dorsal vertebral (D11) (Fig.4) and lumbar spines (L1,L3). There was also an intra-abdominal involvement with a large ‘doughnut-like’ lesion in the liver with subdiaphragmatic metastases (Fig.5). Unfortunately, the patient refused further investigation and defaulted treatment.

## Discussion

Primary tumors of the temporal bone account for about only 1% of all head and neck neoplasms (6). Metastases of malignant tumors to the temporal bone are even rarer and can be overshadowed by mastoiditis symptoms (2,3). The most common malignancies giving rise to temporal bone metastases are from the breast, lung, kidney and gastrointestinal system (2,3,5).

Collet-Sicard syndrome is a rare condition, clinically resulting in paralysis of the vocal cords, palate, and trapezius and sternocleidomastoid muscles (4). This case report demonstrates another rare cause of Collet-Sicard syndrome. Generally, the primary tumor is at an advanced stage by the time temporal bone metastases are clinically detected.

A literature search reveals the most common clinical symptoms in patients with secondary malignant tumors of the temporal bone to be facial palsy followed by symptoms such as hearing loss and otalgia (6–8). In contrast, primary adenocarcinoma of the temporal bone typically has a lengthy clinical course; i.e. a long history of otitis media with otorrhea, otalgia and hearing loss (9). The House-Brackmann facial nerve grading system is widely used to characterize the degree of facial paralysis. In this scale, grade I is assigned to normal function, and grade VI represents complete paralysis. Intermediate grades vary according to function at rest and with effort. The most common otologic symptoms that manifest with facial nerve paralysis are often due to mastoid infection. 

The patient in this report demonstrated features suggestive of extensive mastoiditis on high resolution CT scan of the temporal bone. Subsequently, her general condition deteriorated with progressive difficulty in swallowing and hoarseness of the voice. Histopathological diagnosis revealed metastatic adenocarcinoma of the mastoid, after which a search for the primary site was performed using ^18^F-FDG PET-CT. The findings from the ^18^F-FDG PET-CT study demonstrated extensive lesions with multilevel involvement of the posterior and anterior elements of the spine, the liver and subdiaphragmatic regions. There were no significant changes in the ^18^F-FDG PET-CT study involving the lungs, bowel, breasts or kidneys to suggest primary involvement of these organs. 

Temporal bone imaging is important in the diagnosis of primary as well as secondary neoplasms (8,10). Radiologically, there is widespread erosion of the bone with variable soft tissue lesion. It is difficult to make distinction between these tumors on the basis of these radiologic CT findings (9,10). Whole body functional imaging PET-CT is another imaging modality for the detection of metastatic temporal bone malignancy. Our patient was initially diagnosed as having mastoiditis, and later the clinical impression was revised to metastatic adenocarcinoma upon histopathological specimen analysis. Metastatic carcinoma of the temporal bone needs extensive analysis to search for primary lesion (10–12). Diagnosis relies on appropriate imaging studies, biopsy and immunohistochemistry (11). Several studies have demonstrated the value of immunohistochemical studies involving the differential expression of CK7 and CK20 as a valuable diagnostic biomarker in differentiating primary and metastatic adenocarcinoma (12). The treatment modality consists of a combination of surgery, systemic chemotherapy and radiotherapy. Generally, the prognosis of malignant disease of the temporal bone remains poor (10,11,13).

## Conclusion

Clinical presentation of the metastatic carcinoma of the temporal bone can be overshadowed by infective or inflammatory conditions. This case report emphasizes the point that a high index of clinical suspicion is necessary for the early diagnosis of this aggressive disease which carries a relatively poor prognosis. This case illustrates the usefulness of ^18^F-FDG PET-CT in the instant detection of distant metastases, with the potential to modify management strategy.
